# Have we been overlooking a critical confounder? Time to address adverse childhood experiences in nutrition and chronic disease epidemiology

**DOI:** 10.1017/S1368980025101031

**Published:** 2025-09-05

**Authors:** Marion Tharrey

**Affiliations:** Independent Researcher, Ubud, Bali, Indonesia

**Keywords:** Adverse childhood experiences, Nutritional epidemiology, Chronic diseases, Confounding, Dietary behaviours

Recent advances in public health nutrition research have made significant contributions to understanding how dietary patterns and alcohol consumption shape the risk of chronic diseases. Researchers have made rigorous efforts to account for potential confounding variables, such as socioeconomic position, educational attainment and lifestyle behaviours, to isolate the true effects of diet-related exposures on health. However, one critical and pervasive determinant has remained largely overlooked: adverse childhood experiences (ACEs). Defined as experiences of abuse, neglect, or exposure to household dysfunction before the age of 18^(1)^, ACEs are now widely recognised as important determinants of long-term health, significantly increasing the risk of numerous unhealthy behaviours across the life course^(2)^.

Within the field of public health nutrition, the relationship between ACEs and dietary behaviours remains an emerging area of investigation. Yet, a growing body of evidence suggests that ACEs are associated with unhealthy eating patterns, emotional eating and poor diet quality in adulthood. Cross-sectional analysis of over 30 000 adults in the Southern Community Cohort Study (USA) revealed a significant association between ACEs and lower diet quality, as measured by the Healthy Eating Index-2010, independent of race, sex and income^(3)^. In the context of nutrition-related diseases, low fruit and vegetable intake alongside high consumption of energy-dense, nutrient-poor foods such as sugary, salty and fatty products has long been emphasised as a major public health concern. Using data from the Behavioral Risk Factor Surveillance System (BRFSS, USA), recent evidence indicates that individuals with higher ACE scores are at increased risk of lower overall fruit and vegetable intake, while evidence for vegetable intake specifically remains inconsistent^(4,5)^.

Furthermore, findings from the nationally representative longitudinal Add Health study (USA) revealed that exposure to four or more ACEs was associated with higher consumption of fast food and sugar-sweetened beverages, with these associations partially mediated by college educational attainment and perceived stress^(6)^. Longitudinal evidence has also consistently linked ACEs with food insecurity, with risk increasing alongside cumulative ACE exposure and persisting even after adjustment for key socio-demographic and psychosocial factors^(7,8)^.

Beyond dietary quality, an expanding body of research has implicated ACEs in the development of both eating disorders and alcohol use disorders. A review of seventy studies involving over 300 000 participants found that adverse life experiences, most of which were ACEs, are a significant risk factor for obesity and binge eating disorder, with positive associations reported in more than 85 % of studies. The review also highlighted key psychological and neurobiological mediators, including post-traumatic stress disorder (PTSD), depression, trait anger, perceived stress, body dissatisfaction, dissociation, insecure attachment and neurobiological alterations^(9)^. Childhood food neglect, in particular, has been identified as a specific ACE with long-term implications, increasing the risk of eating disorders such as anorexia nervosa and binge eating disorder, even after adjusting for financial difficulties and other adverse experiences during childhood^(10)^. Emerging research also highlights the association between ACEs and symptoms of ultra-processed food addiction in adulthood^(11,12)^. Regarding alcohol use disorders, a meta-analysis of thirty-seven studies involving over 253 000 participants reported that individuals with four or more ACEs faced a substantially higher risk of harmful alcohol-related behaviours in adulthood. The strength of association varied by severity, with moderate odds for heavy alcohol use and notably stronger odds for problematic alcohol use, reinforcing a clear dose–response relationship^(2)^.

In addition to food-related behavioural outcomes, ACEs have been established as an independent risk factor for a broad range of long-term health conditions and poor health outcomes^(2,13)^. In particular, a systematic review and meta-analysis of cross-sectional studies found that exposure to multiple ACEs was associated with a 46 % higher likelihood of obesity in adulthood^(14)^, and several meta-analyses have further documented associations between ACEs and type 2 diabetes^(15)^, CVD and cancer^(2)^, as well as mental health disorders such as depression^(2,16)^. These health consequences are rarely isolated; they often interact and cluster, compounding risk and burden over the life course. Notably, each additional ACE has been associated with a nearly 13 % increase in the odds of developing multimorbidity in adulthood^(17)^. Biological mechanisms proposed include chronic hypothalamic–pituitary–adrenal axis dysregulation, systemic inflammation and maladaptive stress responses^(18)^. Together, these findings highlight the extensive and enduring impact of early-life adversity on physical and mental health, reinforcing the importance of addressing ACEs within the framework of chronic disease prevention and nutrition research.

Despite meeting the fundamental criteria for a confounder – being associated with both the exposure (nutrition behaviours) and the outcome (chronic diseases), without lying on the direct causal pathway – childhood abuse remains largely unmeasured and unaccounted for in large epidemiological studies in the field of public health nutrition. A growing body of research now provides compelling evidence that individuals exposed to ACEs represent a particularly vulnerable subgroup, more likely to engage in unhealthy eating and drinking behaviours and to face a higher burden of adverse health outcomes. The failure to account for this key determinant may result in residual confounding, potentially distorting estimates of the associations between dietary or alcohol exposures and chronic disease risk. Beyond methodological concerns, recognising this vulnerable population also offers important opportunities for targeted public health interventions aimed at reducing health inequalities and improving dietary behaviours in ACE-exposed populations.

The potential magnitude of this oversight is substantial. According to the WHO, approximately 400 million children under the age of 5 years’ experience regular physical punishment and/or psychological violence from parents or caregivers. Additionally, it is estimated that one in five women and one in seven men report having endured sexual abuse during childhood^(19)^. A recent meta-analysis of data from over 500 000 adults across twenty-two countries converges with these estimates, reporting that approximately 60 % of adults, mostly from high-income countries, experienced at least one ACE before the age of 18^(20)^.

Given the substantial prevalence and lasting health impacts of childhood abuse, I urge public health nutrition researchers to consider its potential confounding role in observational studies. Including validated ACE measures or proxy indicators in cohort studies, national health and nutrition surveys and secondary analyses could improve the accuracy of risk estimates linking nutrition exposures to chronic disease. Furthermore, this adjustment would reflect a growing commitment within public health to address trauma-informed care and its relevance to long-term health trajectories.

It is time for public health nutrition research to formally recognise childhood abuse as a significant, underacknowledged confounder influencing both behaviour and disease outcomes.
